# Placebo response in pharmacological trials in patients with functional dyspepsia—A systematic review and meta‐analysis

**DOI:** 10.1111/nmo.14474

**Published:** 2022-09-27

**Authors:** Michelle Bosman, Fabiënne Smeets, Sigrid Elsenbruch, Jan Tack, Magnus Simrén, Nicholas Talley, Bjorn Winkens, Ad Masclee, Daniel Keszthelyi

**Affiliations:** ^1^ Division of Gastroenterology and Hepatology, Department of Internal Medicine, School of Nutrition and Translational Research in Metabolism (NUTRIM) Maastricht University Medical Center Maastricht The Netherlands; ^2^ Department of Medical Psychology and Medical Sociology, Faculty of Medicine Ruhr University Bochum Bochum Germany; ^3^ Department of Neurology, University Hospital Essen University of Duisburg‐Essen Essen Germany; ^4^ Translational Research Center for Gastrointestinal Disorders (TARGID) Catholic University of Leuven Leuven Belgium; ^5^ Division of Gastroenterology and Hepatology University Hospitals Leuven Leuven Belgium; ^6^ Department of Molecular and Clinical Medicine, Institute of Medicine Sahlgrenska Academy, University of Gothenburg Gothenburg Sweden; ^7^ Centre for Functional GI and Motility Disorders University of North Carolina Chapel Hill North Carolina USA; ^8^ NHMRC Center of research Excellence in Digestive Health University of Newcastle Newcastle New South Wales Australia; ^9^ Department of Methodology and Statistics Care and Public Health Research Institute Faculty of Health, Medicine, and Life Sciences Maastricht University Maastricht The Netherlands

**Keywords:** FD, functional dyspepsia, meta‐analysis, placebo, placebo response

## Abstract

**Background:**

Pharmacological trials in functional dyspepsia (FD) are associated with high placebo response rates. We aimed to identify the magnitude and contributing factors to the placebo response.

**Methods:**

We conducted a systematic review and meta‐analysis including randomized controlled trials (RCTs) with a dichotomous outcome in adult patients with FD that compared an active pharmacotherapeutic treatment with placebo. Our main outcome was identification of the magnitude of the pooled placebo response rate for the following endpoints: symptom responder, symptom‐free responder, adequate relief responder, and combined endpoint responder (i.e., the primary endpoint of each specific trial regarding treatment response). Several putative moderators (i.e., patient, disease, and trial characteristics) were examined.

**Key Results:**

We included 26 RCTs in our analysis. The pooled placebo response rate was 39.6% (95% CI 30.1–50.0) using the symptom responder definition, 20.5% (12.8–31.0) using the symptom‐free responder definition, 38.5% (33.8–43.6) using the adequate relief responder definition, and 35.5% (31.6–39.7) using the combined endpoint responder definition. A lower overall baseline symptom score was significantly associated with a higher placebo response rate. No other moderators were found to significantly impact the placebo response rate. Due to the lack of data, no analyses could be performed according to individual FD subtypes or symptoms.

**Conclusions and Inferences:**

The pooled placebo response rate in pharmacological trials in FD is about 39%, depending on which responder definitions is used. Future trials should consider applying an entry criterion based on minimal level of symptom severity to decrease the placebo response. We also suggest separate reporting of core FD symptoms pending more concrete harmonization efforts in FD trials.

## 1 INTRODUCTION

Functional dyspepsia (FD) is a common disorder of gut‐brain interaction characterized by the presence of various upper gastrointestinal (GI) symptoms, believed to originate from the gastroduodenal region.^1^, ^2^ The estimated prevalence is 9.0% in Western countries and 7.2% worldwide (according to the Rome IV criteria).^3^, ^4^ In addition, FD represents a substantial health burden, being associated with an impaired quality of life, and also with increased healthcare consumption.^3^, ^5^, ^6^, ^7^ The assessment of treatment efficacy in FD is a methodological and clinical challenge, in part due to the lack of standardized methods for symptom reporting.^8^ Despite being recommended by the US Food and Drug Administration (FDA) and the European Medicines Agency (EMA), there are no universally accepted well‐defined patient‐reported outcome measures (PROMs) available for adequate evaluation of treatment efficacy in FD.^8^, ^9^, ^10^, ^11^, ^12^ Increasing knowledge about factors influencing treatment responses, including the role of the placebo response, is necessary for both research and clinical settings in order to improve therapeutic outcomes.

Previous trials in disorders of gut‐brain interaction, including FD, have shown that there is a substantial proportion of patients that benefit from a placebo intervention.^13^ The magnitude of the placebo response has profound effects on the outcomes of these trials and causes a generally low assay sensitivity (i.e., the ability of a trial to successfully differentiate between an efficacious and an inefficacious treatment). The placebo effect has been identified as a phenomenon influenced by multiple factors related to the psychosocial treatment context, including patient and disease characteristics, as well as trial characteristics of randomized clinical trials (RCTs).^13^ A recent meta‐analysis gave further insight in the placebo response rate in patients with irritable bowel syndrome (IBS),^14^ where the data suggested that not the individual patient characteristics but rather trial characteristics primarily determine the magnitude of the placebo response in pharmacological trials, with implications for the design of future RCTs in IBS. As far as FD is concerned, a recent meta‐analysis has examined the placebo response in a broad range of therapies for FD, demonstrating a pooled placebo response rate of 32.4%^15^ However, this meta‐analysis included RCTs with different types of active therapy, including herbal preparations and food supplements, and placebo response rates ranged from 0.0% to 84.5%. Including pharmacological agents only could lead to more homogeneous results, which is of particular relevance for improving trial design and endpoint definitions. In addition, some specific predictors of the placebo response in FD, that is, higher body mass index (BMI), nonsmoking status, low symptom severity at baseline, symptom progression during run‐in, and a longer study period,^15^, ^16^, ^17^ have been identified by previous studies. However, these findings are in need of an independent validation.^13^

Examining factors driving the placebo response in pharmacological trials in FD is therefore an important step forward in developing standardized clinical trial designs in order to minimize the magnitude of the placebo response and hence increase the likelihood of demonstrating the effectiveness of pharmacological treatment. Therefore, we conducted a systematic review and meta‐analysis with the aim to characterize the pooled placebo response rate in pharmacological RCTs of FD and to identify possible moderators of the placebo response rate.

## 2 MATERIALS AND METHODS

This systematic review and meta‐analysis were performed in accordance with the guidance provided by the Cochrane Handbook for Systematic Reviews of Interventions^18^ and PRISMA.^19^ The protocol is registered in PROSPERO (CRD42020176958).

### 2.1 Search strategy and selection criteria

MEDLINE, EMBASE, and the Cochrane Central Register of Controlled Trials were screened using the following search strategy: (dyspepsia [as medical subject heading and free‐text term] “OR” functional dyspepsia [as free‐text term]) “AND” (placebo “OR” placebo effect [both as medical subject heading and free‐text terms]). Studies were selected between May 1964 and December 2021, with English set as filter. In addition, reference lists of eligible studies were hand‐screened for potential additional studies. Corresponding authors of the studies were contacted if the full‐text article was not available.

Eligible trials were RCTs that examined the effect of pharmacological therapies compared with a placebo (as pill/tablet/capsule but not a liquid formulation) in adult patients (≥18 years) with the diagnosis of FD (based on either symptom‐based diagnostic criteria [i.e., Rome criteria] or physician's assessment) without any macroscopic abnormalities observed at upper gastrointestinal endoscopy. A minimum treatment duration was required of 28 days in which active therapy was given. Trials had to report a patient‐reported dichotomous outcome of response to therapy.^9^ The first period of cross‐over trials was eligible for inclusion if the authors provided data prior to crossover. We excluded trials with treatments other than therapies based on a singular pharmacological agent (e.g., multi‐component herbal preparations including peppermint oil^20^ and food supplements, as expectations regarding treatment efficacy can differ substantially between these therapies versus drug therapy^9^). We also excluded trials that analyzed the effect of *H. pylori* eradication (as these are associated with a late‐onset control of symptoms^1^). Moreover, we excluded trials that reported only on treatment satisfaction as the outcome^21^, trials that duplicated or reanalyzed previously obtained trial data, and any publication type that was not a full‐text article (as data in an article without full text was considered incomplete and therefore insufficient in detail for the planned analyses).

The search, the assessment of title and abstract (according to the predefined eligibility criteria), and subsequently the full‐text assessment of all potentially relevant studies were all independently conducted by the same two researchers (FS and MB). A medical librarian was contacted to supervise the search. The disagreement between the investigators was resolved by discussion with a third investigator (DK).

### 2.2 Data analysis

The primary outcome assessed was the magnitude of the placebo response rate. We distinguished between a symptom responder (i.e., response to therapy based on reporting an improvement on a continuous scale in symptom score by the patient), a symptom‐free responder (i.e., response to therapy based on patients reporting they experience no more symptoms), an adequate relief responder (i.e., response to therapy based on dichotomous reporting of an improvement on an adequate relief‐type question by the patient), and a pooled endpoint. The adequate relief responder was divided into a binary (yes/no) adequate relief question responder and a Likert‐scale‐based adequate relief question responder. The pooled endpoint (i.e., the combined endpoint responder) was used for the moderator analyses and included the primary endpoint of each specific trial regarding treatment response, either a symptom responder, a symptom‐free responder, or an adequate relief responder, as the trials were generally powered for their specific primary endpoints. In case of multiple eligible primary endpoints, both were included, with the main primary outcome as indicated by the authors being included in the combined responder endpoint. If the primary endpoint was ineligible due to a continuous outcome, then a dichotomous secondary endpoint was used, but only as long as the continuous primary outcome measure also concerned treatment efficacy. Other eligible secondary endpoints were not included as the power calculation of each trial was based on primary endpoints. Secondary dichotomization of data reported in a continuous fashion was not considered sensible as that would have made responder rates in the individual trials arbitrary by nature, contributing to an unnecessary increase in uncertainty of the pooled results. Response to the active intervention was also assessed along the same criteria.

The secondary outcome assessed was the effect of various trial, patient, and disease characteristics (i.e., moderators) on the pooled placebo response rate, according to the combined responder definition. The moderators assessed (see Table S1) were identified from previous research on this subject and were extracted when available.^14^, ^16^, ^17^ Varying baseline symptom scores scales were standardized to a 0–10 rating scale (see Table S2). For all included trials, data were extracted independently (FS and MB) into a Microsoft Excel spreadsheet as dichotomous outcomes (responder versus non‐responder). Data were extracted as intention‐to‐treat analyses, with dropouts assumed to be treatment failures, wherever trial reporting allowed this.^18^

### 2.3 Assessment of bias risk

Two researchers (FS and MB) used the Cochrane Risk of Bias Tool independently to assess the risk of bias at the individual study level.^22^ Disagreements were resolved by discussion with a third investigator (DK). Bias was assessed as a judgment (low, unclear, or high risk of bias) for six domains of bias (sequence generation, allocation concealment, blinding, incomplete outcome data, selective outcome reporting, and other sources of bias). Criteria for the judgment of high risk of bias were either one domain with high risk of bias or four domains with unclear risk of bias.^22^ Studies with a high risk of bias were then excluded from further assessment.

### 2.4 Statistical analysis

Data were stored and analyzed using R version 4.0.1 (Vienna, Austria).^23^ Data were pooled from the placebo arms of the clinical trials using a random‐effects model based on logit transformation, allowing for any heterogeneity between trials. Separate analyses were run to evaluate the pooled placebo response rate across all studies for the different responder definitions, with a 95% confidence interval (CI). Data were presented in forest plots, where funnel plots were included for detecting publication bias. Similar analyses were also conducted for the intervention arms of the clinical trials. To obtain the therapeutic gain, the difference between the intervention response rate and placebo response rate was calculated separately for each trial and subsequently pooled using a random‐effects model. We considered to perform a within‐study comparison of the definitions of responders, however, this was not feasible because multiple responder definitions were only defined in four of all studies.

Each moderator was examined in a separate meta‐regression model rather than in a combined model to avoid overfitting of data and listwise deletion (i.e., some moderators are measured in one study but not in another study). Linearity has been checked using scatterplots. Missing data were extracted as non‐reported with an exception regarding the run‐in period and the exclusion of patients with IBS‐symptoms, where the assumption was made that no run‐in period had been conducted and that patients were not excluded when they had IBS‐symptoms if no information about this was provided. Results were expressed in odds ratios with a 95% confidence interval (CI). Heterogeneity was evaluated using *I* ^2^‐statistics (interpreted as low (<25%), moderate (25%–50%), and high (>50%) heterogeneity) and homogeneity was evaluated using the *Q*‐statistics (with *p* ≤ 0.10 considered statistically significant).^24^ For moderation analysis, considering the potential increase in number of false positive results, correction for multiple testing using the false discovery rate (FDR) method was applied.^25^ A *p*‐value of ≤0.05 was considered statistically significant.

## 3 RESULTS

**FIGURE 1:**
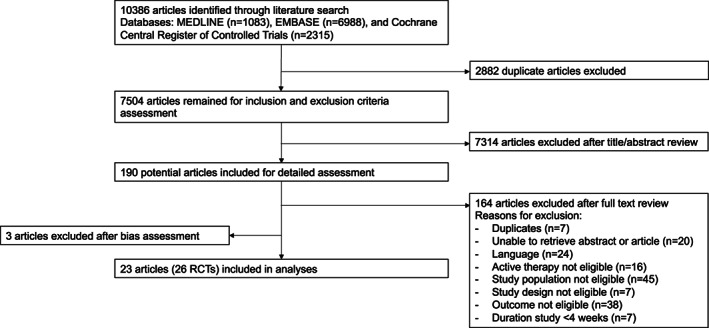
PRISMA (Preferred Reporting Items for Systematic Reviews and Meta‐analysis Analysis) flow diagram of trials identified, excluded, and included in the meta‐analysis.

The search generated 10.386 articles, 190 of which appeared to be relevant based on title and/or abstract and were retrieved for further assessment (Figure 1). Of these, 164 were excluded for various reasons, leaving 26 articles that met inclusion criteria. Three^26^, ^27^, ^28^ were subsequently excluded because of high risk for bias. Eventually 26 RCTs, described in 23 articles^29^, ^30^, ^31^, ^32^, ^33^, ^34^, ^35^, ^36^, ^37^, ^38^, ^39^, ^40^, ^41^, ^42^, ^43^, ^44^, ^45^, ^46^, ^47^, ^48^, ^49^, ^50^, ^51^ (i.e., 3 articles^39^, ^40^, ^43^ have each described 2 RCTs), were included in the systematic review and meta‐analysis. Detailed characteristics of individual trials are provided in the supplementary (Tables S2 and S3). Bias assessment is reported in the supplementary (Figure S1). Most of the included trials had a low risk of bias (19 of the 26 included RCT's).

**FIGURE 2:**
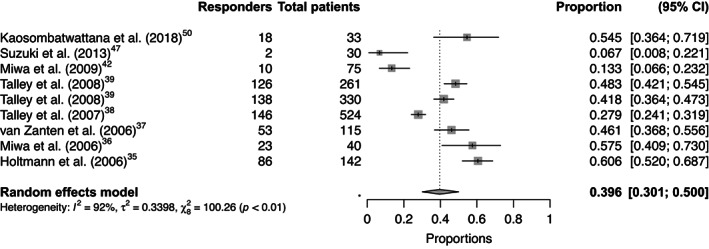
Forest plot of the proportion of placebo responders with the symptom responder definition (9 trials with this endpoint).

**FIGURE 3:**
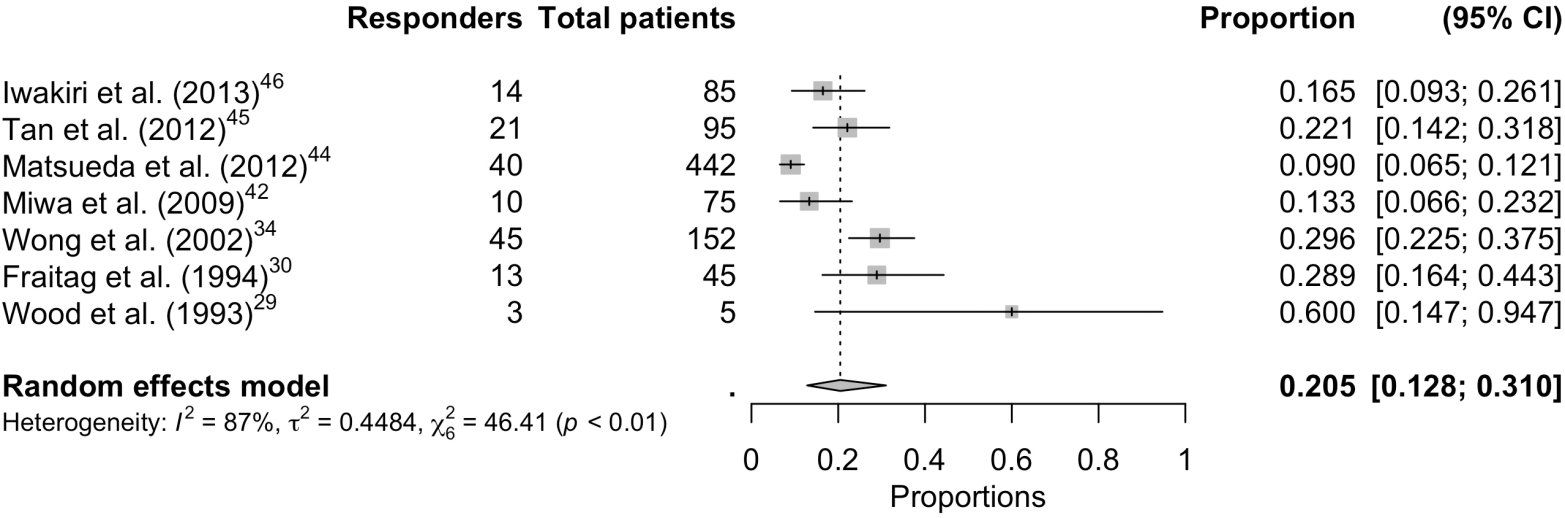
Forest plot of the proportion of placebo responders with the symptom‐free responder definition (7 trials with this endpoint).

**FIGURE 4:**
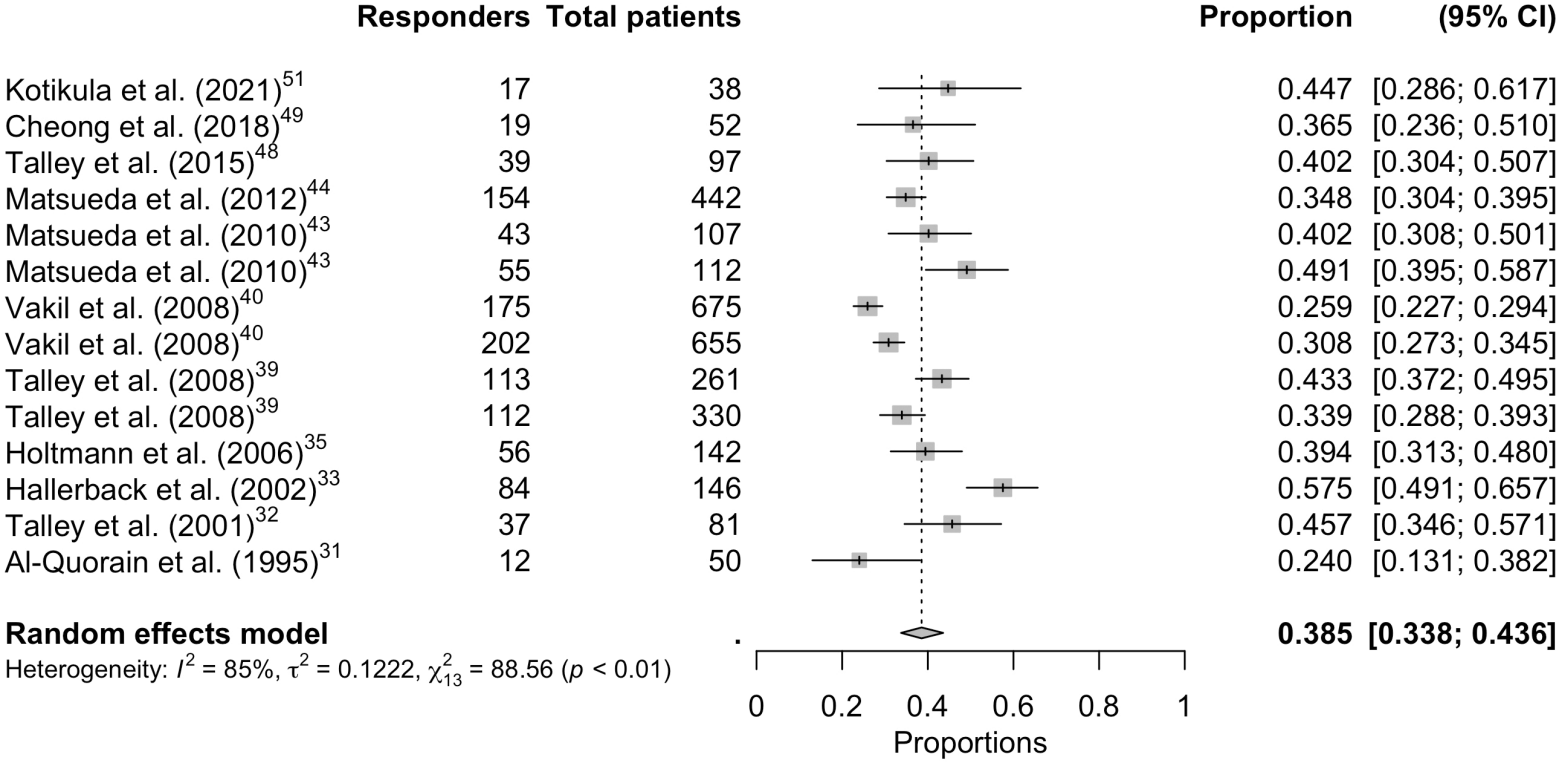
Forest plot of the proportion of placebo responders with the adequate relief question responder definition (14 trials with this endpoint).

**FIGURE 5:**
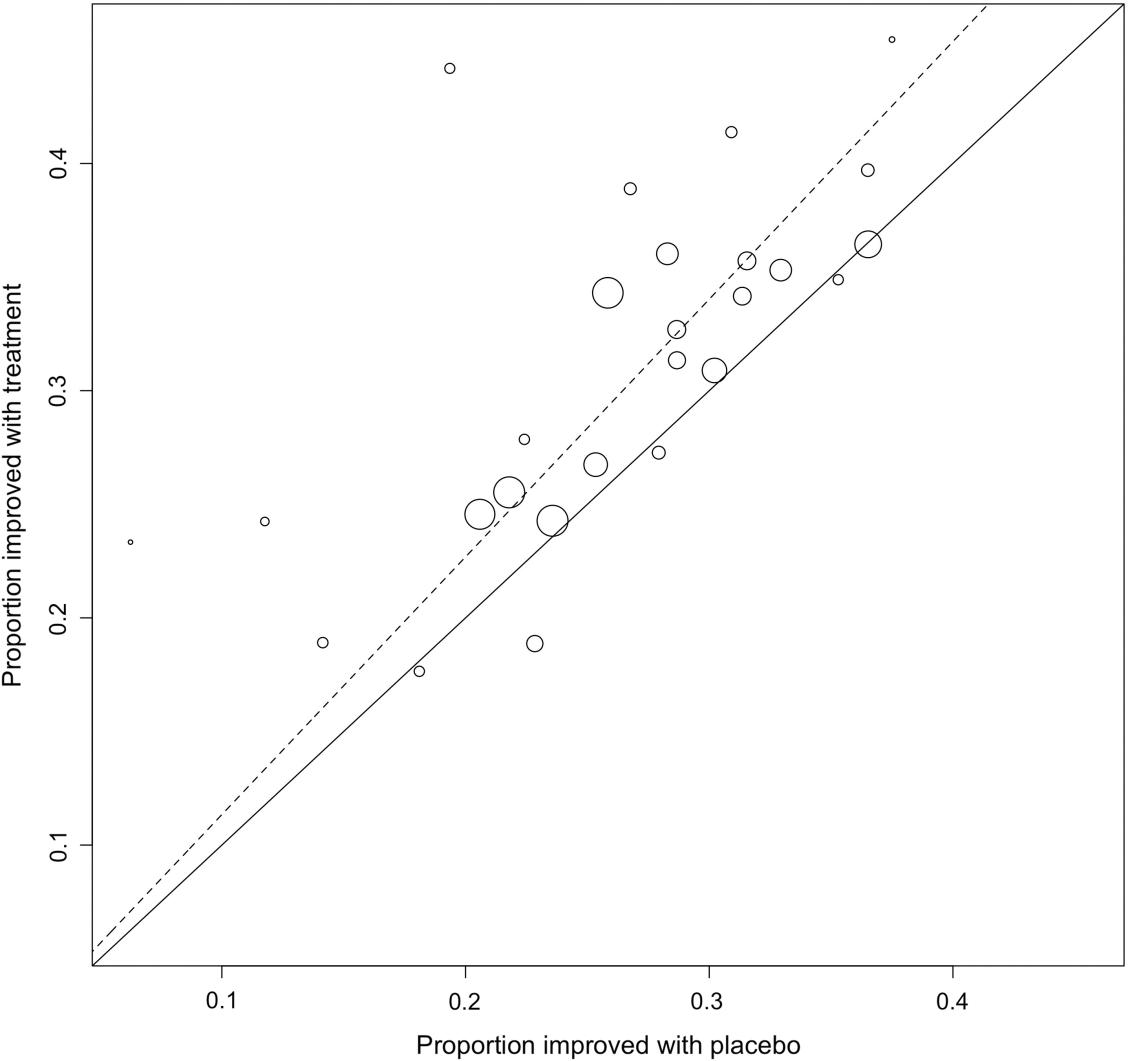
L'Abbe plot of the response rate (defined as the combined endpoint responder) in the intervention group against the response rate in the placebo group.**The equality line (solid line) and overall effect line (dashed line) are shown. Circle size represents sample size.

Overall pooled placebo and intervention response rates according to the symptom responder (Figure 2 and Figure S2), symptom‐free responder (Figure 3 and Figure S3), adequate‐relief responder (Figure 4 and Figure S4), and combined endpoint responder (Figures S5 and S6) are reported in Table 1. Figure 5 shows the response rates in the intervention group against the response rates in the placebo group as defined by the combined endpoint responder.

**TABLE 1:** Placebo response rate, intervention response rate, and the therapeutic gain of the primary outcome assessment.

	Number of trials with this variable	Placebo		Intervention		Therapeutic gain (95% CI)
		Response rate (95% CI)	*I* ^2^ (95% CI)	Response rate (95% CI)	*I* ^2^ (95% CI)	
Symptom responder	9	39.6% (30.1–50.0)	92.0% (90.5–99.2)	49.6% (38.4–60.8)	95.4% (85.0–98.4)	7.9% (4.5–11.4)
Symptom‐free responder	7	20.5% (12.8–31.0)	87.1% (60.8–98.0)	26.0% (19.5–33.7)	84.2% (68.6–99.3)	5.1% (0.0–11.1)
Adequate relief responder	14	38.5% (33.8–43.6)	85.3% (69.0–94.4)	50.5% (44.2–56.8)	93.6% (89.5–98.3)	10.7% (5.3–16.1)
Binary (yes/no) question	7	39.3% (30.8–48.6)	90.6% (73.2–97.7)	49.1% (38.9–59.4)	95.1% (88.6–99.2)	6.3% (1.1–11.5)
Likert‐scale‐based question	7	38.3% (33.8–43.1)	65.5% (18.3–96.0)	51.6% (45.0–58.1)	88.0% (82.8–99.1)	14.0% (4.5–23.5)
Combined endpoint responder	26	35.5% (31.6–39.7)	84.6% (83.5–96.2)	44.9% (39.8–50.2)	93.3% (92.2–97.9)	8.7% (4.9–12.4)

Nine trials^35^, ^36^, ^37^, ^38^, ^39^, ^42^, ^47^, ^50^ provided data for the outcome assessment of proportion of patients who were symptom score responders. The placebo response rate ranged from 6.7% to 60.6%, with an overall pooled placebo response rate of 39.6% (95% CI 30.1%–50.0%), with substantial heterogeneity between the trials (*I* ^2^ 92.0%). The pooled intervention response rate for this endpoint was 49.6% (95% CI 38.4%–60.8%), with substantial heterogeneity between the trials (*I* ^2^ 95.4%), resulting in a therapeutic gain of 7.9% (95% CI 4.5%–11.4%). Seven trials^29^, ^30^, ^34^, ^41^, ^44^, ^45^, ^46^ provided data for the outcome assessment of proportion of patients who where symptom‐free responders. The placebo response rate ranged from 9.0% to 60.0%, with an overall pooled placebo response rate of 20.5% (95% CI 12.8%–31.0%), with substantial heterogeneity between the trials (*I* ^2^ 87.1%). The pooled intervention response rate for this endpoint was 26.0% (95% CI 19.5%–33.7%), with substantial heterogeneity between the trials (*I* ^2^ 84.2%), resulting in a therapeutic gain of 5.1% (95% CI 0.0%–11.1%). Fourteen trials^31^, ^32^, ^33^, ^35^, ^39^, ^40^, ^43^, ^44^, ^48^, ^49^, ^51^ provided data for the outcome assessment of proportion of patients who were overall‐treatment‐effect question responders. The placebo response rate ranged from 24.0% to 57.5%, with an overall pooled placebo response rate of 38.5% (95% CI 33.8%–43.6%), with substantial heterogeneity between the trials (I^2^ 85.3%). The pooled intervention response rate for this endpoint was 50.5% (95% CI 44.2%–56.8%), with substantial heterogeneity between the trials (*I* ^2^ 93.6%), resulting in a therapeutic gain of 10.7% (95% CI 5.3%–16.1%). Out of these fourteen trials, seven trials^32^, ^33^, ^40^, ^48^, ^49^, ^51^ provided data for the outcome assessment of proportion of patients who were a binary question responder with a pooled placebo response rate of 39.3% (95% CI 30.8%–48.6%), and seven trials^31^, ^35^, ^39^, ^43^, ^44^ provided data for the outcome assessment of proportion of patients who were a Likert‐scale question responder with a pooled placebo response rate of 38.3% (95% CI 33.8%–43.1%).

As for trial, patient and disease characteristics as categorical moderators, none were associated with the magnitude of the placebo response (Table 2). As for the numeric variables as moderators, we found a significant association between the overall baseline symptom score and the placebo response rate (*p* = 0.039), indicating that patients with lower overall baseline symptom scores were more likely to have a higher placebo response (Table 3). There was no significant association between the placebo response rate and the other numeric variables (Table 3).

**TABLE 2:** Moderators of categorical variables of the placebo response rate for the combined endpoint responder definition.

	Number of trials with this variable	Pooled placebo response rate (95% CI)	*I* ^2^	Combined *I* ^2^	*Q* statistics	Odds ratio (95% CI)	*p* value	Corrected p value (using FDR)
**Categorical variable**								
Year of publication^a^								
2006 or earlier	9	41.6% (33.5–50.2)	79.35%	89.38%	2.458	1.44 (0.91–2.26)	0.117	0.652
2007 or later	17	33.4% (29.1–38.1)	84.63%					
Geographical location								
Europe	5	45.2% (34.7–56.2)	74.53%	87.15%	2.360	1.75 (0.86–3.56)	0.125	0.542
Asia	13	32.4% (25.8–39.7)	82.85%					
America	2	43.4% (36.9–50.2)	0%	88.32%	0.892	1.61 (0.60–4.32)	0.345	0.538
Asia	13	32.4% (25.8–39.7)	82.85%					
Trial setting								
Single center	6	40.8% (32.0–50.2)	69.38%	89.76%	1.107	1.33 (0.78–2.26)	0.293	0.672
Multicenter	20	34.6% (29.9–39.5)	87.33%					
Primary	4	45.0% (27.7–63.7)	93.81%	89.50%	1.712	1.51 (0.81–2.81)	0.191	0.677
Secondary/tertiary	22	34.7% (30.6–39.0)	82.29%					
Run‐in period								
No	12	36.6% (30.1–43.6)	82.38%	90.22%	0.043	1.05 (0.67–1.64)	0.837	0.882
Yes	14	35.2% (30.1–40.8)	86.56%					
Duration run‐in period								
<2 weeks	5	39.1% (27.9–51.5)	90.91%	88.75%	1.049	1.33 (0.77–2.28)	0.306	0.628
≥2 weeks	9	31.9% (27.7–36.4)	67.04%					
Placebo run‐in								
No	10	37.9% (32.1–44.1)	89.19%	87.77%	3.851	1.85 (1.001–3.42)	0.050	0.650
Yes	4	24.7% (16.1–35.9)	50.32%					
Proportion of pt assigned to placebo								
Less than 50%	10	37.1% (30.0–45.0)	87.60%	90.14%	0.175	1.10 (0.70–1.73)	0.676	0.775
Approximately 50%	16	35.0% (30.0–40.3)	83.85%					
Exlcusion of patients with IBS symptoms								
No	9	39.3% (32.4–46.7)	74.26%	90.08%	0.9071	1.25 (0.79–1.98)	0.341	0.578
Yes	17	34.2% (29.4–39.3)	87.03%					
Mean age								
≤40 years	7	36.8% (30.0–44.1)	77.93%	90.08%	0.907	1.25 (0.79–1.98)	0.341	0.605
>40 years	18	34.2% (29.5–39.3)	84.29%					
Sex								
<70% female	15	37.2% (31.9–42.8)	85.41%	89.68%	0.197	1.11 (0.71–1.74)	0.657	0.776
>70% female	11	33.8% (28.0–40.2)	80.17%					
Duration of FD‐symptoms/diagnosis								
>5 years	3	41.7% (32.5–51.4)	52.66%	91.74%	0.533	1.38 (0.58–3.28)	0.465	0.605
≤5 years	5	33.2% (24.6–43.0)	89.15%					
BMI								
≥25 kg/m^2^	6	33.1% (27.6–39.0)	84.24%	92.79%	1.312	1.46 (0.76–2.81)	0.252	0.655
<25 kg/m^2^	6	25.4% (17.9–34.7)	86.43%					
H. pylori infection								
No	4	38.9% (33.0–45.1)	50.98%	91.15%	0.541	1.33 (0.62–2.82)	0.462	0.621
Yes	12	33.0% (25.6–41.5)	89.46%					
Use of Rome criteria								
No	6	38.7% (25.8–53.4)	90.57%	90.05%	0.287	1.16 (0.67–2.01)	0.592	0.721
Yes	20	35.3% (31.1–39.8)	83.58%					
Rome criteria								
II	14	35.5% (30.7–40.6)	84.67%	91.09%	0.989	1.42 (0.71–2.84)	0.320	0.624
III	4	26.7% (13.8–45.2)	87.45%					
Level of bias								
Unclear	7	46.0% (35.1–57.2)	75.15%	88.23%	4915	1.72 (1.07–2.79)	0.027	0.527
Low	19	33.4% (29.6–37.5)	83.20%					
Dose								
Three times a day	8	38.7% (32.2–45.5)	80.62%	89.95%	0.467	1.18 (0.73–1.92)	0.494	0.621
Once or twice a day	18	34.7% (29.8–40.1)	85.29%					
Escalating dose								
Yes	3	42.4% (35.7–49.3)	10.41%	90.31%	0.714	1.34 (0.68–2.65)	0.398	0.575
No	23	35.1% (30.8–39.7)	86.25%					
Active therapy								
Antidepressants	6	34.3% (23.2–47.5)	84.93%	90.35%	0.903	1.54 (0.63–3.74)	0.342	0.556
PPI	5	26.7% (18.4–37.0)	85.57%					
5HT4‐agonist	3	36.9% (24.2–51.7)	96.11%	95.83%	1.096	1.74 (0.62–4.90)	0.295	0.639
PPI	5	26.7% (18.4–37.0)	85.57%					
Prokinetics	5	37.1% (30.9–43.9)	61.81%	86.69%	1.858	1.61 (0.81–3.21)	0.173	0.675
PPI	5	26.7% (18.4–37.0)	85.57%					
Prokinetics	5	37.1% (30.9–43.9)	61.81%	86.46%	0.097	1.12 (0.54–2.34)	0.755	0.818
Antidepressants	6	34.3% (23.2–47.5)	84.93%					

Abbreviations: BMI, body mass index; FD, functional dyspepsia; FDR, false discovery rate; IBS, irritable bowel syndrome; PPI, proton pomp inhibitor.

^a^ Corresponding to the publication of the Rome III criteria, which was used as a surrogate marker of the process of increased scientific rigor of clinical trial conduct and quality of reporting.

**TABLE 3:** Moderators with numeric variables of the placebo response rate for the combined endpoint responder definition.

Numerical variable	Number of trials with this variable	*I* ^2^	*Q* statistics	Odds ratio (95% CI)	*p* value	Corrected *p* value (using FDR)
Study size of placebo group	26	89.17%	1.181	1.00 (0.998–1.001)	0.277	0.675
Duration of therapy (in weeks)	26	90.11%	0.933	1.05 (0.95–1.15)	0.334	0.620
Proportion of female	26	89.59%	0.143	0.71 (0.12–4.27)	0.705	0.786
Proportion of side effects	9	87.88%	0.736	3.13 (0.23–42.24)	0.391	0.587
Proportion of dropouts	23	90.52%	1.688	6.68 (0.38–117.35)	0.194	0.631
Study visits	20	87.02%	2.378	0.89 (0.76–1.03)	0.123	0.600
Number of centers	20	88.85%	0.0001	1.00 (0.99–1.01)	0.991	0.991
Overall baseline symptom score	12	83.46%	11.723	**0.66 (0.53–0.84)**	**0.001**	**0.039**

Bold indicates significant *p*‐value of ≤0.05.

Abbreviation: FDR, false discovery rate.

## 4 DISCUSSION

In this meta‐analysis describing 26 FD pharmacological RCTs, the magnitude of the placebo response rate was 35.5% using the combined endpoint responder definition, 39.6% using the symptom responder definition, 20.5% using the symptom‐free responder definition, and 38.5% using the adequate relief question responder definition. Studies with patients reporting a lower overall baseline symptom score were significantly associated with a higher placebo response rate.

This meta‐analysis was prompted by the fact that only very limited data are available regarding the pooled placebo response rate and its possible moderators in pharmacological trials in FD. The pooled placebo response rates in this meta‐analysis were in line with the pooled placebo response rate in another recent meta‐analysis.^15^ However, approximately half of the included articles in that meta‐analysis had a non‐pharmacological active therapy and varying dosage forms, what probably led to more heterogeneity in their results with larger confidence intervals. In this meta‐analysis, with a more rigorous and stringent selection (i.e., single pharmacological agents, exclusion of *H. pylori* eradication studies, and careful assessment of bias with the exclusion of studies with high risk of bias) results are more specific and relevant for future trials demonstrating the effectiveness of a pharmacological treatment. In addition, we have assessed different definitions of responder than in the aforementioned meta‐analysis. Therefore, our meta‐analysis complements earlier work and provides important insight to inform future drug development in FD, in particular power calculations on which to base projected sample sizes.

It is noteworthy that there was considerable heterogeneity across trials regarding responder definitions. A valid reason for this is the fact that there are no universally validated and accepted PROMs for assessment of treatment efficacy in FD clinical trials.^8^ Despite this, we found that the pooled placebo response rates and the pooled intervention rates were nearly equal for the different therapy responder outcome definitions, with the exception of the symptom‐free responder endpoint, where both placebo and intervention responses were considerably lower. This can be explained by that fact that the criteria for the responder definition for the symptom‐free responder endpoint are considerably stricter, making the criteria for the symptom‐free responder definition less likely to be achieved with lesser improvement in symptoms than the other responder definitions.^9^

The previously identified predictors of the placebo response rate, that is, an association of a higher BMI,^16^, ^17^ a nonsmoking status,^16^ low symptom severity at baseline,^16^ symptom progression during run‐in,^16^ and a longer study period^15^ with a higher placebo response, were not completely in line with the results of this meta‐analysis. The effect of smoking status and symptom progression during run‐in could not be evaluated, because of incomplete reporting of these factors in the individual trials. In general, several other subject factors (including diet, exercise, and stress) were incompletely reported, despite the fact that dietary and lifestyle factors are frequently reported by patients with FD in relation to their symptoms.^52^ Our results did not show a significant association between the study duration and the pooled placebo response rate. Previous findings on the effect of this possible moderator on the placebo response rate in different disorders of the gut‐brain interaction have shown incongruent results, which could be related to the natural fluctuation of FD and varying study visits with different study durations.^13^, ^14^, ^53^, ^54^ Contrary to the prior findings, we did not find a significant effect of BMI on the pooled placebo response rate. Nonetheless, previous studies have shown a relationship between the patients' BMI and certain upper GI symptoms and disorders of gut‐brain interaction.^55^, ^56^, ^57^, ^58^ However, these associations and the influence of the patients' BMI on the placebo response are still in need of further investigation.^55^, ^56^, ^57^, ^58^

We did find that a lower overall baseline symptom score was significantly associated with a higher pooled placebo response rate. This association is in line with previous observations in FD,^16^, ^40^ however, previous findings of this association in other disorders are incongruent.^14^, ^54^, ^59^, ^60^ We speculate that this association can be explained by the fact that less severe symptoms are more likely to decrease as part of spontaneous remission due to the natural fluctuation of FD, while patients with more severe symptoms would be less likely to respond because they could represent a more resistant group. In addition, modification of symptom perception by patients' cognitions and expectations may be involved. There was no significant association between baseline symptom scores for specific FD symptoms and the placebo response, however, these specific symptoms were only reported in a small number of trials, which made a moderation analysis unreliable (see Table S4).

Currently, the Rome IV criteria divides the umbrella term “FD” into three subtypes (i.e., post‐prandial distress syndrome, epigastric pain syndrome, and overlap syndrome).^2^, ^3^, ^61^ However, we were unable to assess the influence of FD subtype on the placebo response, due to changing definitions of FD subtypes with the different Rome criteria, in addition to the lack of consequent reporting in trials.^2^, ^61^ Given these findings, and the fact that the FDA recommends that the effect of treatment should be based on each individual symptom,^8^, ^9^, ^10^ it is concerning to observe that the overall symptom score and specific symptom scores for FD core symptoms (i.e., postprandial fullness, early satiation,^62^ epigastric pain, epigastric burning, and upper abdominal bloating^63^) were inadequately reported, and if reported, many different questionnaires and rating scales were used.

In line with findings in other disorders of gut‐brain interaction,^13^, ^14^, ^59^ there were no other patient or disease characteristics significantly associated with the pooled placebo response. This suggest that the division between placebo “responders” and “non‐responders” does not depend on the individuals' characteristics.^14^ Meanwhile, patient expectations are still important patient‐specific determinants of the placebo response.^13^, ^14^, ^54^ Neither did we find associations between trial characteristics and the pooled placebo response, contrary to previous findings in other GI disorders (including disorders of gut‐brain interaction).^13^, ^14^, ^59^

The strengths of this study include the rigorous selection criteria, separate assessment of different study endpoints for the primary outcome assessment, and the detailed assessment of various moderators. There are limitations to be mentioned. First and foremost, the results of any meta‐analysis rely on the reporting and quality of the included trials, even when adhering to strict inclusion criteria. The fact that no significant effect of moderators, other than baseline symptom severity, was identified is possibly related to the limited data available from individual trials. Together with the lack of standardized methods for trial design, entry criteria (differences in Rome criteria), responder definitions, methods for symptom assessment, and reporting on contextual factors, this causes substantial heterogeneity between trials. Pooling of results for purposes of the analysis are therefore inherently difficult. Hence, we decided to analyze different responder definition endpoints separately for the primary outcome (to create some harmonization between the trials to perform our analysis) and to perform analysis according to the primary outcomes for the moderator analysis, for which the particular trial was powered for. However, given this apparent lack of standardized reporting strategies, any such categorization chosen for the purposes of meta‐analyses will inherently carry arbitrarily and potentially contribute to the uncertainty of findings. Second, some commonly used pharmacological agents for FD (i.e., PPI and H2 blockers) were represented in a smaller number in this analysis due to the exclusion of several articles. This was due to certain trial characteristics, that is, that duration of therapy was <4 weeks and patients with no previous diagnosis of FD were included. Third, case definitions also varied across trials, with the majority of trials using Rome II and only one trial using the most recent Rome IV definition, which might also have influenced results. Last, seven of the 26 trials were at unclear risk of bias, which may have influenced results.

In conclusion, the pooled placebo response rate in pharmacological trials in FD is about 39% for the different responder definitions, with the exception of the symptom‐free responder definition (with a pooled placebo response rate of 20.4%). We found that less severe baseline symptoms were associated with an increased placebo response rate. Therefore, future trials should consider introducing an entry criterion based on minimal level of symptom severity, which can easily be determined during a run‐in period. This is in line with FDA recommendations for IBS trials^64^ that suggest the selection of patients with sufficient symptom intensity to make demonstration of a clinically meaningful improvement possible. Pending more concrete harmonization efforts in FD trial design, we recommend the reporting of the core symptoms of FD individually, taking the evolution of the definitions for FD and its subtypes and potential future modifications hereof into account, in order to enhance comparability across trials. In general, a more adequate assessment and reporting in the future of different variables, including those related to patient expectations, is needed in order to establish firm recommendations regarding their effect on placebo response.

## AUTHOR CONTRIBUTIONS

Guarantor of the article: Michelle Bosman, MD.

DK conceptualized the review project and FS developed the study protocol under the supervision of DK. MB and FS did the literature search, screened, and reviewed all the published literature, and did the data extraction. MB did the data analysis. MB and DK did the data interpretation and MB drafted the manuscript and prepared the tables and figures. SE, JT, MS, NT, AM, and DK provided an important and substantial intellectual contribution and a critical revision of the manuscript. BW provided a critical analysis of the data analysis. MB and DK have verified the underlying data. DK supervised all study phases. All authors approved the final version of the manuscript before its submission. All authors had full access to all the data in the study and have accepted responsibility to submit for publication.

## FUNDING INFORMATION

None to declare.

## CONFLICT OF INTEREST

Part of the work of MHMA is financed by EU grant H2020 DISCOvERIE/848228. SE reports grants from Deutsche Forschungsgemeinschaft (German Research Foundation), outside the submitted work. JT reports personal fees from Adare, Arena, Christian Hansen, Devintec, Ironwood, Shire, Truvion, Abbott, and Menarini; and grants from Shire, Tsumura, Sofar, Mylan, outside the submitted work. MS reports grants and personal fees from Danone Nutricia Research, and Glycom; personal fees from Nestlé, Ironwood, Menarini, Biocodex, Arena, Adnovate, Shire, Tillotts, Kyowa Kirin, Takeda, Alimentray Health, AlfaSigma, and Falk Foundation; and grants from Genetic Analysis, outside the submitted work. NT reports personal fees from Allakos, Aviro Health, Antara Life Sciences, Arlyx, Bayer, Danone, Planet Innovation, Takeda, Viscera Labs, twoXAR, Viscera Labs, Dr Falk Pharma, Censa, Cadila Pharmaceuticals, Progenity Inc, Sanofi‐aventis, Glutagen, ARENA Pharmaceuticals, IsoThrive, BluMaiden, HVN National Science Challenge; non‐financial support from HVN National Science Challenge NZ; funding form the National Health and Medical Research Council for the Centre for Research Excellence in Digestive Health; holds an NHMRC Investigator grant; has a patent Biomarkers of IBS licensed (#12735358.9–1405/2710383 and [#12735358.9–1405/2710384]), Licensing Questionnaires Talley Bowel Disease Questionnaire licensed to Mayo/Talley, Nestec European Patent licensed, Singapore Provisional Patent NTU (Ref: TD/129/17 “Microbiota Modulation Of BDNF Tissue Repair Pathway” issued), copyright Nepean Dyspepsia Index (NDI) 1998 and Editorial: Medical Journal of Australia (Editor in Chief), Up to Date (Section Editor), Precision and Future Medicine, Sungkyunkwan University School of Medicine, South Korea, Med (Journal of Cell Press); participates committees Australian Medical Council (AMC) Council Member (2016–2019), MBS Review Taskforce (2016–2020), NHMRC Principal Committee, Research Committee (2016–2021), Asia Pacific Association of Medical Journal Editors (APAME) (current), GESA Board Member (2017–2019); and Misc: Avant Foundation (judging of research grants) (2019), community and patient advocacy groups Advisory Board IFFGD (International Foundation for Functional GI Disorders) and AusEE; outside the submitted work. AM reports grants from ZonMw, Will Pharma, Allegan, Grünenthal, Pentax Europe, and the Dutch Cancer Society; and financial compensation to their institution for giving scientific advice to Bayer, Kyowa Kirin, and Takeda, outside the submitted work. DK reports research funding from Grunenthal, Allergan, Will Pharma, UEG, MLDS, Rome Foundation, ZonMw, and Horizon 2020, and has received speaker's fee (paid to host institution) from Dr Falk., outside the submitted work. FS and BW declare no competing interests. No funding of interests to declare.

## Supporting information


Table S1
Table S2
Table S3
Table S4
Figure S1
Figure S2
Figure S3
Figure S4
Figure S5
Figure S6
