# Supplementary material for: Placebo response in pharmacological trials in patients with functional dyspepsia—A systematic review and meta‐analysis
Source: Neurogastroenterol Motil. 2022 Sep 27;35(2):e14474. doi: 10.1111/nmo.14474 (PMC10078497; doi:10.1111/nmo.14474)
Supplement: Supplementary file 1 — Table S1 Table S2 Table S3 Table S4 Figure S1 Figure S2 Figure S3 Figure S4 Figure S5 Figure S6 [file NMO-35-0-s001.pdf]

Supporting Information

Systematic review with meta-analysis: placebo response in pharmacological trials in patients with functional dyspepsia

Michelle Bosman, MD, Fabiënne Smeets, MD, PhD, Sigrid Elsenbruch, PhD, Jan Tack, MD, PhD, Magnus Simrén, MD, PhD, Nicholas Talley, MD, PhD, FRACP, FAHMS, Bjorn Winkens, PhD, Ad Masclee, MD, PhD, Daniel Keszthelyi, MD, PhD.

Supplementary Table 1. Data extracted about various trial-, patient- and disease characteristics for each trial as secondary outcome assessment.

FD = Functional Dyspepsia, IBS = Irritable Bowel Syndrome, BMI = Body Mass Index.

**Trial- and drug characteristics**	**Patient- and disease characteristics**
Year of publication	Mean age
Geographical setting	Sex
Trial setting: single versus multicenter	Race
Trial setting: primary versus secondary/tertiary care	BMI
Study design: parallel versus cross-over	Subtype of FD
Run-in phase: duration	Mean duration of diagnosis/symptoms
Run-in phase: use of placebo	H. Pylori status: actual infection
Study size	H. Pylori status: prior infection
Randomization ratio	Substance use (alcohol, tabaco, coffee)
Criteria used to define FD	Baseline symptom scores
Questionnaires used for symptom assessment	Exclusion of patients with IBS symptoms
Type of active therapy	
Duration of therapy	
Dosing schedule	
Number of face-to-face visits	
Proportion of side effects	
Proportion of dropouts	

Supplementary Table 2. Detailed study characteristics of the endpoints and overall baseline symptom scores used in the individual studies.

NR = Not reported, PPI = proton pomp inhibitor

	Study	Primary treatment efficacy endpoint	Secondary treatment efficacy endpoint†	Second primary treatment efficacy endpoint	Symptom questionnaire	Scale	Baseline overall symptom score	Baseline overall symptom score: 11-point scale
**Prokinetics**	Talley et al. (2008)	Adequate relief responder (likert)	-	Symptom responder	Leeds dyspepsia questionnaire	0-40	13.5 (±4.0)	3.62
		Adequate relief responder (likert)	-	Symptom responder	Leeds dyspepsia questionnaire	0-40	13.6 (±4.1)	3.65
	Vakil et al. (2008)	-	Adequate relief responder (binary)	-	Symptom questionnaire	NR	-	-
		-	Adequate relief responder (binary)	-	Self-report questionnaire	NR	-	-
	Holtmann et al. (2006)	Adequate relief responder (likert)	-	Symptom responder	Leeds Dyspepsia Questionnaire	0-40	12.1 (±6.4)	3.25
	Hallerback et al. (2002)	-	Adequate relief responder (binary)	-	Symptom diary	01-49	2.75 (±?)	0.62
	Al-Quorain et al. (1995)	-	Adequate relief responder (likert)	-	Symptom questionnaire	NR	-	-
	Wood et al. (1993)	-	Symptom-free responder	-	Symptom diary	NR	-	-
**PPI**	Suzuki et al. (2013)	Symptom responder	-	-	Likert dyspepsia severity scale	NR	-	-
	Iwakiri et al. (2013)	Symptom-free responder	-	-	Dyspepsia symptom questionnaire	01-56	24.6 (±6.6)	4.83
	Talley et al. (2007)	Symptom responder	-	-	Symptom questionnaire	0-39	9.7 (±?)	2.67
	Van Zanten et al. (2006)	Symptom responder	-	-	Global overall symptom questionnaire	NR	-	-
	Wong et al. (2002)	Symptom-free responder	-	-	Symptom questionnaire	01-60	22.0 (±0.4)	4.03
**Antidepressants**	Kaosombatwattana et al. (2018)	Symptom responder	-	-	Leeds dyspepsia questionnaire	0-40	10.5 (±5.1)	2.82
	Cheong et al. (2018)	Adequate relief responder (binary)	-	-	Dyspepsia symptom score questionnaire	0-31	8.23 (±4.0)	2.83
	Talley et al. (2015)	Adequate relief responder (binary)	-	-	Validated daily symptoms diaries	NR	-	-
	Tan et al. (2012)	Symptom-free responder	-	-	Hong Kong dyspepsia index	01-60	26.51 (±?)	4.86
	Miwa et al. (2009)	Symptom responder	-	-	Symptom questionnaire (modifying GSRS)	01-24	8.44 (±3.7)	3.87
	Van Kerkhoven et al. (2008)	Symptom-free responder	-	-	Self-report questionnaire	0-69	19 (±8.0)	2.99
**Cholinesterase-inhibitors**	Matsueda et al. (2012)	Adequate relief responder (likert)	-	Symptom-free responder	Dyspepsia symptom questionnaire	NR	-	-
	Matsueda et al. (2010)	Adequate relief responder (likert)	-	-	Symptom diary	NR	-	-
		Adequate relief responder (likert)	-	-	Symptom diary	NR	-	-
**Others**	Kotikula et al. (2021)	Adequate relief responder (binary)	-	-	Global overall symptom questionnaire	NR	-	-
	Miwa et al. (2006)	Symptom responder	-	-	Symptom questionnaire	NR	-	-
	Talley et al. (2001)	Adequate relief responder (binary)	-	-	Symptom diary	NR	-	-
	Fraitag et al. (1994)	-	Symptom-free responder	-	Symptom diary	NR	-	-

†The secondary endpoint was included in case the primary endpoint was not eligible (ie, continuous outcome)
‡Varying baseline symptom scores scales were standardized to a 0-10 rating scale: (baseline overall symptom score x 11) / maximum score original scale

Supplementary Table 3. Detailed study characteristics of the individual studies.

NR = Not reported, PPI = Proton-pump inhibitors, IBS = Irritable Bowel Syndrome

	Study	Geographical location	Setting (primary vs. secondary care) (single vs. multi center)	Total patients (%female)	Total patients in placebo group	Randomization ratio	Rome criteria	Exlcusion of patients with IBS symptoms	Patients with H. Pylori actual infection (%)	Active therapy	Duration of therapy (weeks)	No. of daily doses	No. of study visits	Proportion of side effects in placebo group
**Prokinetics**	Talley et al. (2008)	Multicontinental	Secondary multi center	525 (64.7%)	261	1:1	II	Yes	0%	Itopride	8	3	5	NR
		Multicontinental	Secondary multi center	645 (64.2%)	330	1:1	II	Yes	0%	Itopride	8	3	5	NR
	Vakil et al. (2008)	Multicontinental	Secondary multi center	1360 (100%)	675	1:1	II	Yes	NR	Tegaserod	6	1	5	NR
		Multicontinental	Secondary multi center	1307 (100%)	655	1:1	II	Yes	NR	Tegaserod	6	1	5	NR
	Holtmann et al. (2006)	Europe	Secondary multi center	554 (62.8%)	142	1:1:1:1	II	No	21.3%	Itopride	8	3	3	37.3%
	Zhao et al. (2005)	Asia	Secondary single center	43 (60.5%)	13	2:1	II	Yes	NR	Domperidone	8	3	NR	NR
	Hallerback et al. (2002)	Europe	Primary multi center	589 (61.5%)	146	1:1:1:1	-	Yes	31.6%	Mosapride	6	3	3	NR
	Al-Quorain et al. (1995)	Asia	Secondary single center	98 (49.4%)	50	1:1	-	Yes	NR	Cisapride	4	3	3	0%
	Wood et al. (1993)	Europe	Primary multi center	11 (72.7%)	5	1:1	-	Yes	NR	Cisapride	4	3	4	0%
	Chung et al. (1993)	Asia	Secondary single center	29 (NR)	15	1:1	-	No	NR	Cisapride	4	3	2	0%
**PPI**	Majewski et al. (2016)	America	Secondary single center	73 (56.0%)	35	1:1	II	Yes	NR	Esomeprazole	10	1	6	NR
	Suzuki et al. (2013)	Asia	Secondary multi center	53 (68.2%)	30	1:1	III	Yes	32.1%	Lansoprazole	4	1	NR	NR
	Iwakiri et al. (2013)	Asia	Secondary multi center	338 (56.7%)	85	1:1:1:1	III	No	23.1%	Rabeprazole	8	1	5	42.4%
	Talley et al. (2007)	Multicontinental	Primary multi center	1589 (66.7%)	524	2:1	-	Yes	33.7%	Esomeprazole	7	1	5	NR
	Van Zanten et al. (2006)	America	Primary multi center	223 (63.5%)	115	1:1	II	Yes	22.9%	Esomeprazole	8	1	NR	NR
	Wong et al. (2002)	Asia	Secondary multi center	453 (74.0%)	152	1:1:1	II	Yes	38.2%	Lansoprazole	4	1	3	NR
**Antidepressants**	Kaosombatwattana et al. (2018)	Asia	Secondary single center	61 (70.5%)	33	1:1	III	Yes	NR	Nortriptyline	8	1	3	NR
	Cheong et al. (2018)	Asia	Secondary single center	107 (79.4%)	52	1:1	II	No	0%	Imipramine	12	1	5	NR
	Talley et al. (2015)	America	Secondary multi center	292 (75%)	97	1:1:1	II	No	14%	Amitriptyline, escitalopram	10	1	6	20.6%
	Tan et al. (2012)	Asia	Secondary single center	193 (72%)	95	1:1	II	Yes	15.7%	Sertraline	8	1	3	NR
	Miwa et al. (2009)	Asia	Secondary multi center	150 (73.2%)	75	1:1	II	Yes	20.8%	Tandospirone citrate	4	2	NR	NR
	Van Kerkhoven et al. (2008)	Europe	Secondary multi center	160 (59.4%)	80	1:1	-	No	NR	Venlafaxine	8	1	2	NR
**Cholinesterase-inhibitors**	Matsueda et al. (2012)	Asia	Secondary multi center	892 (59.3%)	442	1:1	III	Yes	NR	Acotiamide	4	3	5	60.4%
	Matsueda et al. (2010)	Asia	Secondary multi center	317 (56.2%)	107	1:1:1	II	No	NR	Acotiamide hydrochloride	4	2	NR	22.4%
		Asia	Secondary multi center	451 (65%)	112	1:1:1:1	II	No	NR	Acotiamide hydrochloride	4	2	NR	15.2%
**Others**	Kotikula et al. (2021)	Asia	Secondary single center	72 (73.6%)	38	1:1	IV	Yes	0%	Pregabaline	8	1	3	NR
	Miwa et al. (2006)	Asia	Secondary single center	81 (81.5%)	40	1:1	II	No	43.2%	Rebamipide	4	2	NR	NR
	Talley et al. (2001)	Multicontinental	Secondary multi center	320 (68.8%)	81	1:1:1:1	I	Yes	22.8%	Alosetron	12	2	4	51%
	Fraitag et al. (1994)	Europe	Secondary multi center	146 (53.7%)	45	1:1:1:1	-	No	NR	Fedotozine	6	3	8	NR

Supplementary Figure 1. Risk of Bias assessment according to The Cochrane Risk of Bias Tool.

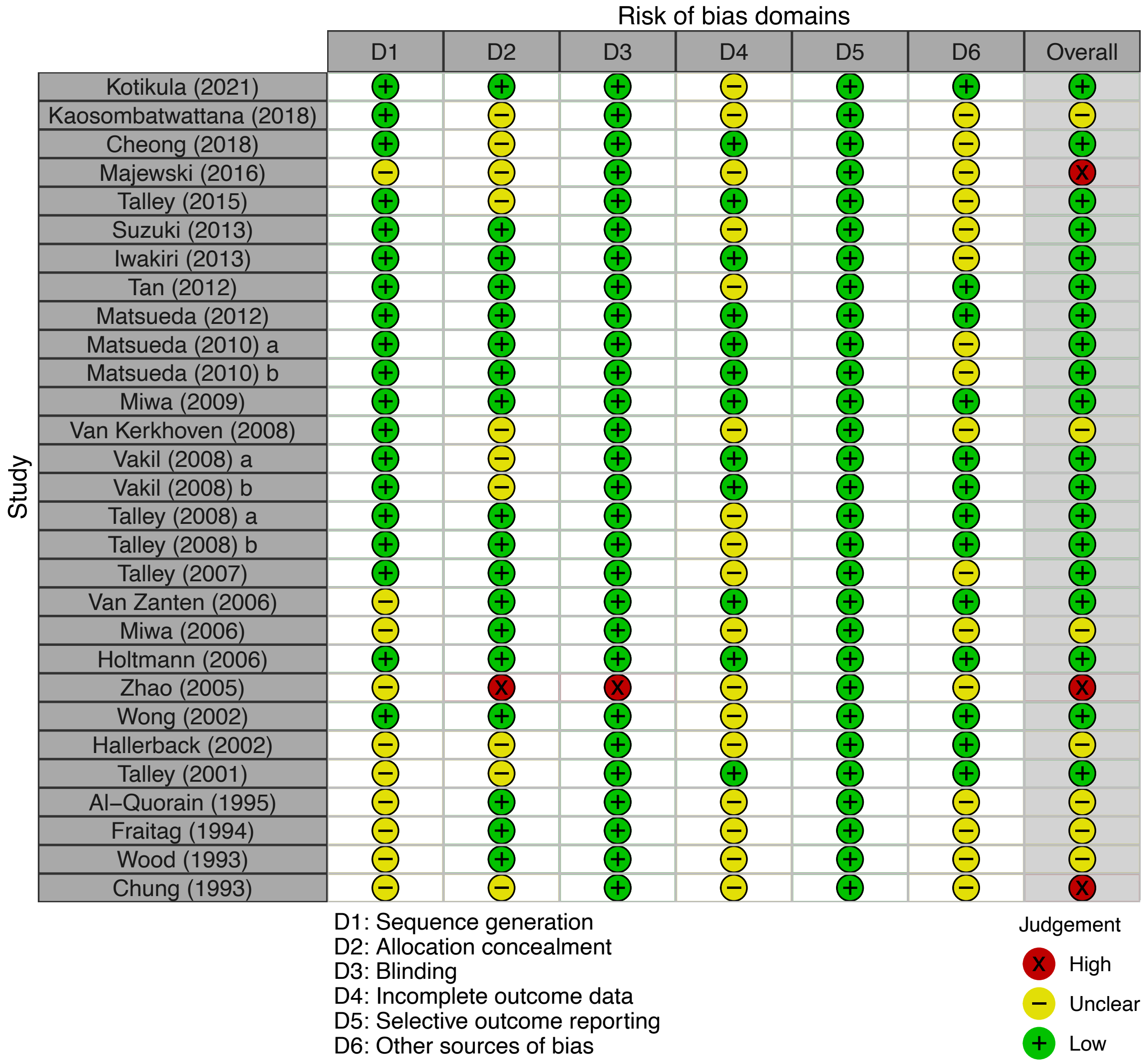

Supplementary Figure 2. Funnel plot of the proportion of placebo responders with the symptom responder definition (9 trials with this endpoint).

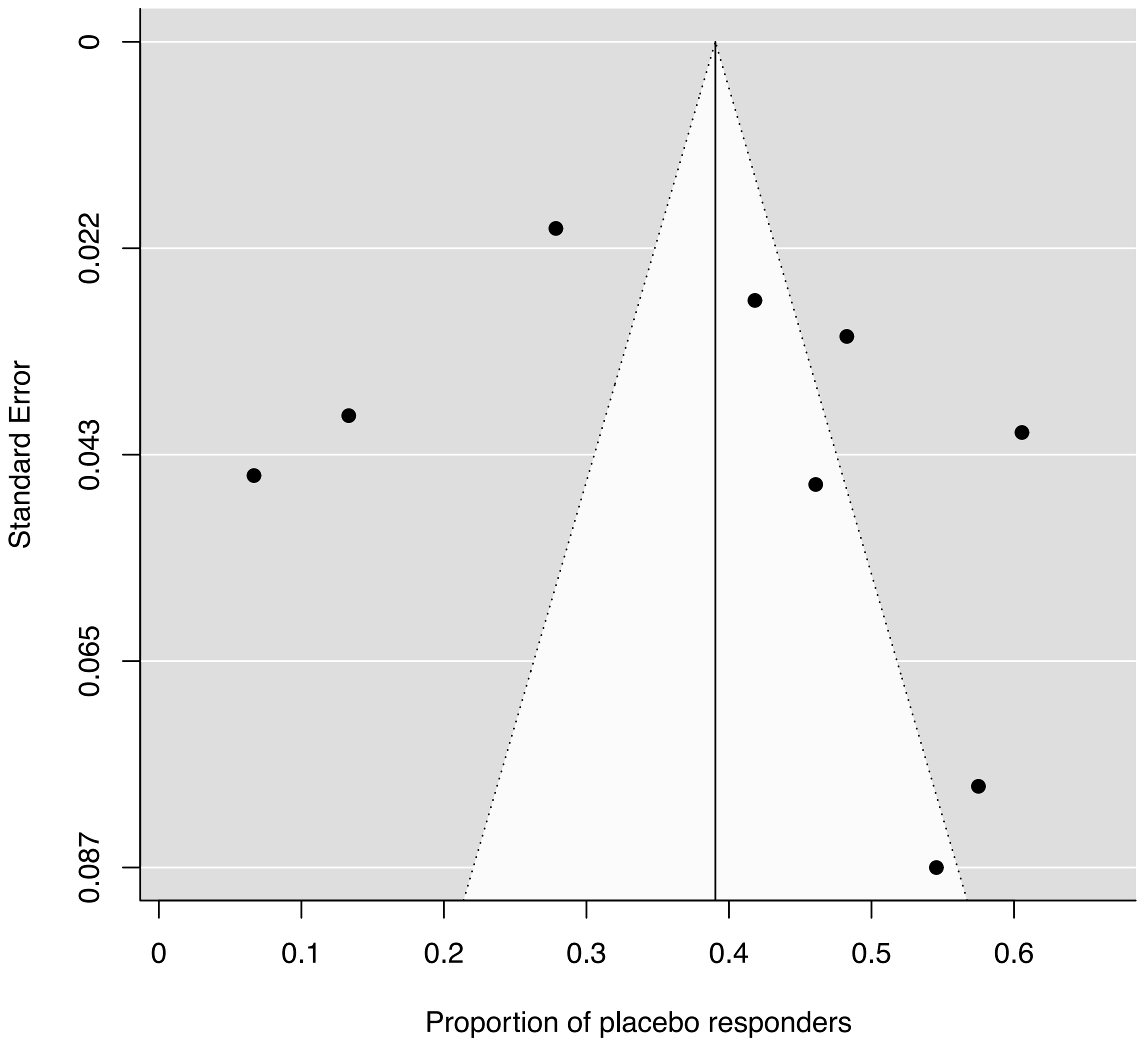

Supplementary Figure 3. Funnel plot of the proportion of placebo responders with the symptom-free responder definition (7 trials with this endpoint).

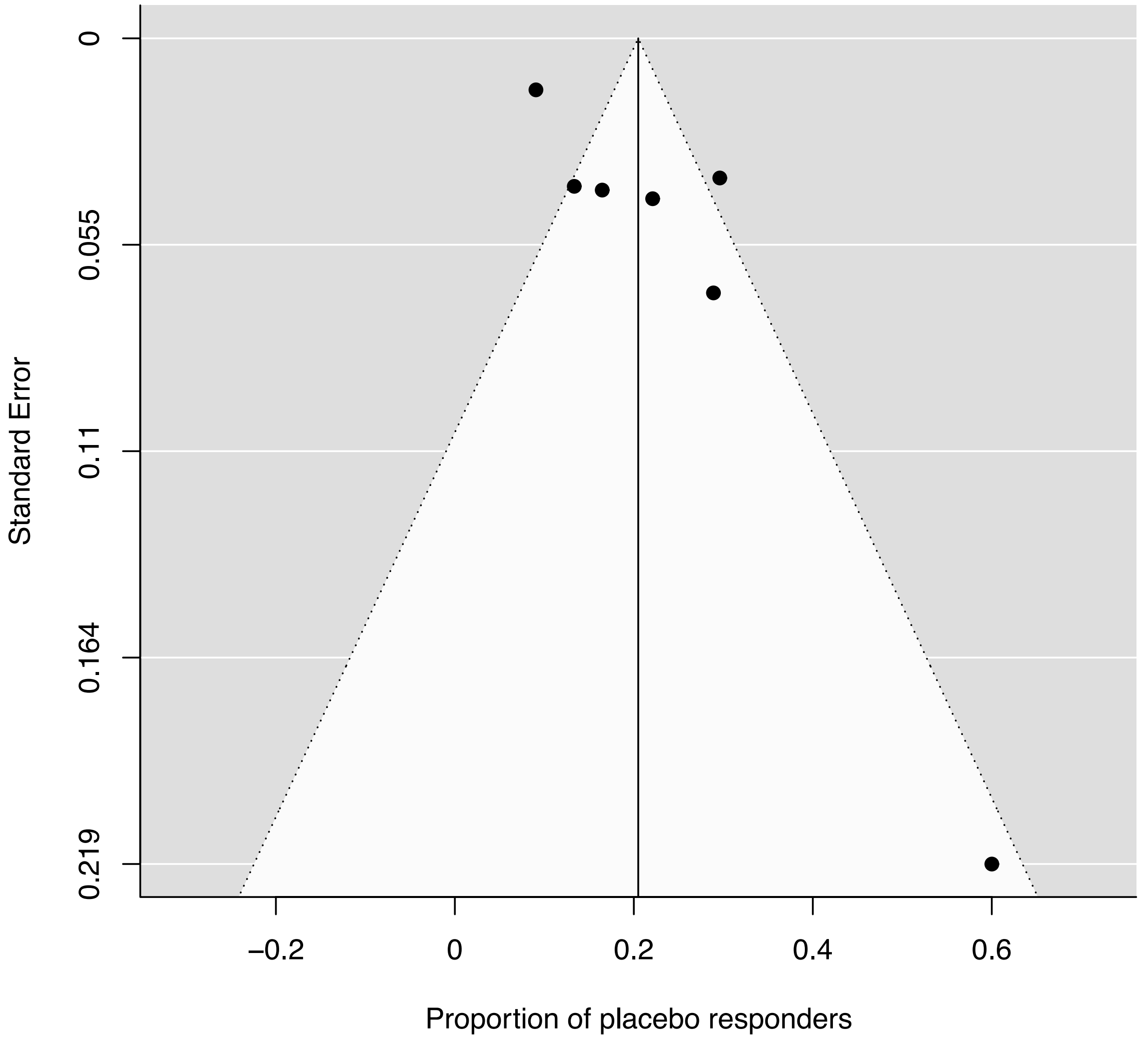

Supplementary Figure 4. Funnel plot of the proportion of placebo responders with the adequate relief question responder definition (14 trials with this endpoint).

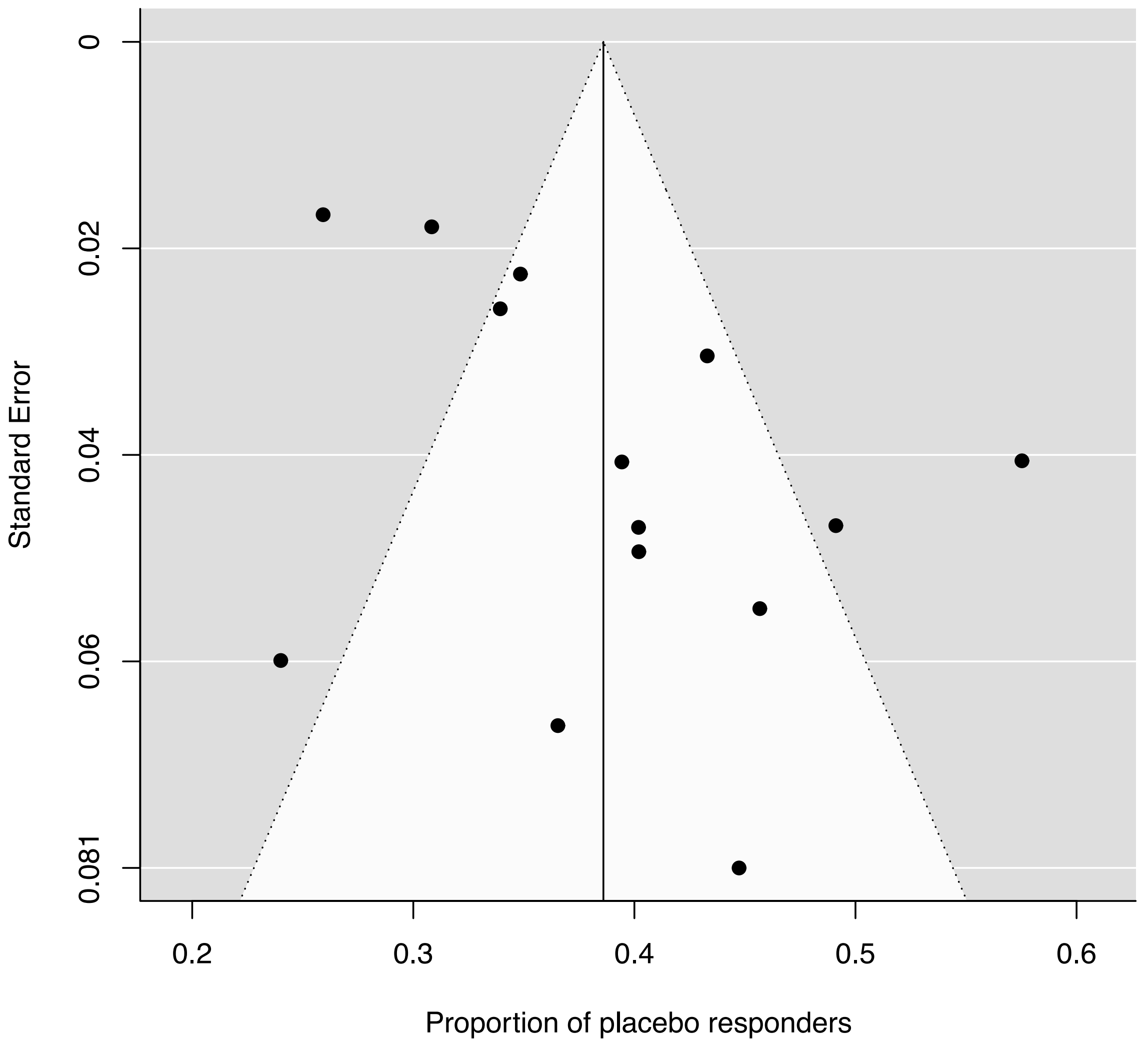

Supplementary Figure 5. Forest plot of the proportion of placebo responders with the combined endpoint responder definition (26 trials with this endpoint).

	Responders	Total patients	Proportion	(95% CI)
Kotikula et al. (2021)[51]	17	38	0.447	[0.286; 0.617]
Kaosombatwattana et al. (2018)[50]	18	33	0.545	[0.364; 0.719]
Cheong et al. (2018)[49]	19	52	0.365	[0.236; 0.510]
Talley et al. (2015)[48]	39	97	0.402	[0.304; 0.507]
Suzuki et al. (2013)[47]	2	30	0.067	[0.008; 0.221]
Iwakiri et al. (2013)[46]	14	85	0.165	[0.093; 0.261]
Tan et al. (2012)[45]	21	95	0.221	[0.142; 0.318]
Matsueda et al. (2012)[44]	154	442	0.348	[0.304; 0.395]
Matsueda et al. (2010)[43]	43	107	0.402	[0.308; 0.501]
Matsueda et al. (2010)[43]	55	112	0.491	[0.395; 0.587]
Miwa et al. (2009)[42]	10	75	0.133	[0.066; 0.232]
van Kerkhoven et al. (2008)[41]	31	80	0.388	[0.281; 0.503]
Vakil et al. (2008)[40]	175	675	0.259	[0.227; 0.294]
Vakil et al. (2008)[40]	202	655	0.308	[0.273; 0.345]
Talley et al. (2008)[39]	113	261	0.433	[0.372; 0.495]
Talley et al. (2008)[39]	112	330	0.339	[0.288; 0.393]
Talley et al. (2007)[38]	146	524	0.279	[0.241; 0.319]
van Zanten et al. (2006)[37]	53	115	0.461	[0.368; 0.556]
Miwa et al. (2006)[36]	23	40	0.575	[0.409; 0.730]
Holtmann et al. (2006)[35]	56	142	0.394	[0.313; 0.480]
Wong et al. (2002)[34]	45	152	0.296	[0.225; 0.375]
Hallerback et al. (2002)[33]	84	146	0.575	[0.491; 0.657]
Talley et al. (2001)[32]	37	81	0.457	[0.346; 0.571]
Al–Quorain et al. (1995)[31]	12	50	0.240	[0.131; 0.382]
Fraitag et al. (1994)[30]	13	45	0.289	[0.164; 0.443]
Wood et al. (1993)[29]	3	5	0.600	[0.147; 0.947]
**Random effects model**		.	**0.355**	**[0.316; 0.397]**

Heterogeneity: $I^2 = 85\%$, $\tau^2 = 0.1568$, $\chi^2_{25} = 162.68$ ($p < 0.01$)

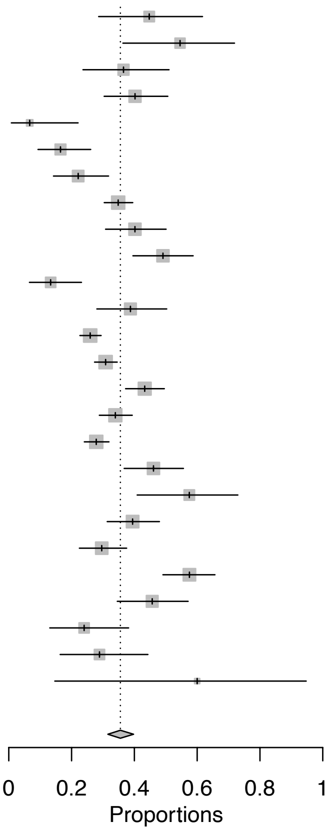

Supplementary Figure 6. Funnel plot of the proportion of placebo responders with the combined endpoint responder definition (26 trials with this endpoint).

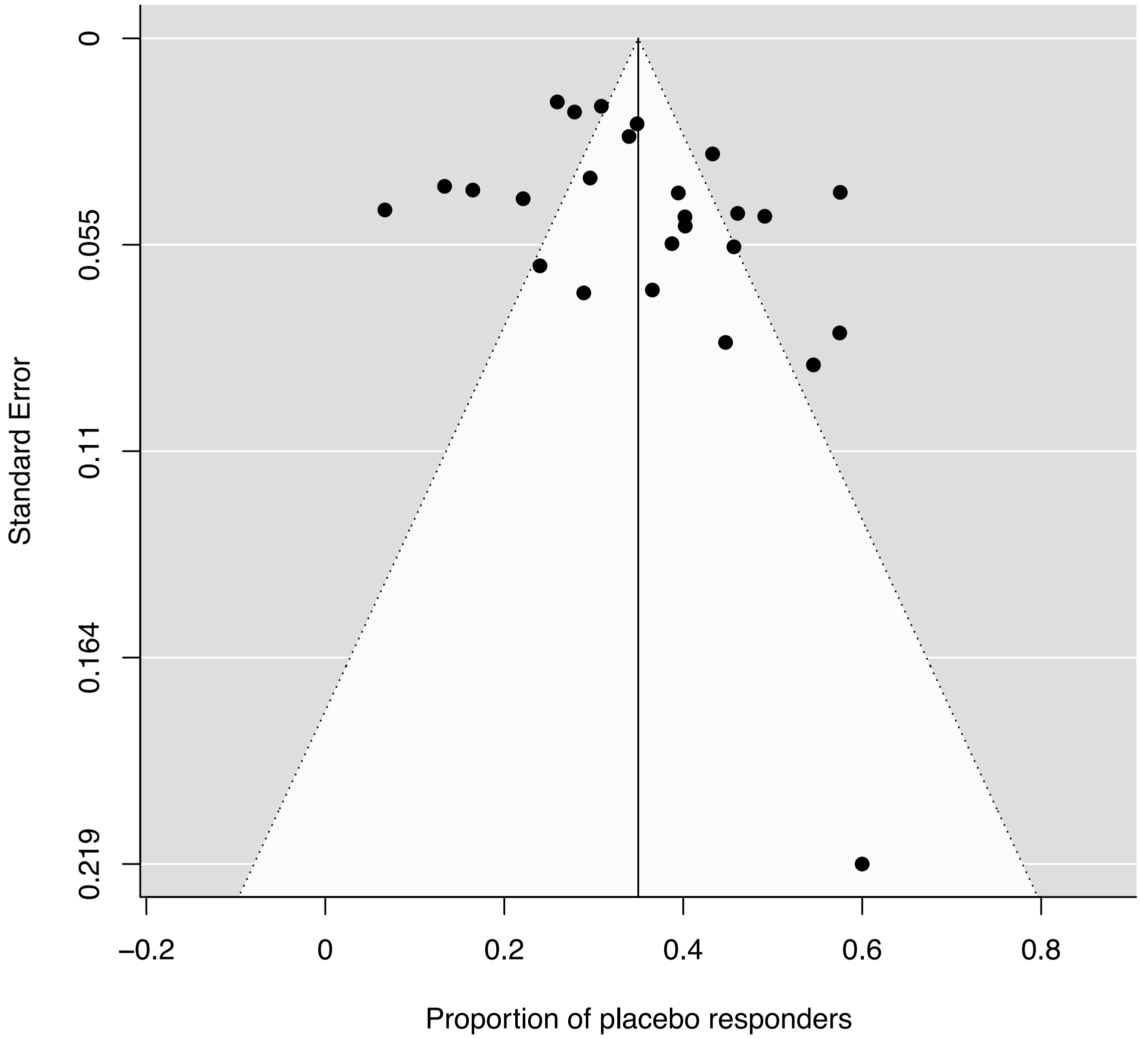

Supplementary Table 4. Baseline symptom scores for specific symptoms as moderators (numeric variables) of the placebo response rate for the combined endpoint responder definition.*

* Bold indicates significant p-value of ≤0.05. FDR = False Discovery Rate

Numerical variable	Number of trials with this variable	Combined I²	Q-statistics	Odds ratio (95% confidence interval (%))	P-value	Corrected p-value (using FDR)
Overall baseline symptom score	12	83.46%	11.723	**0.66 (0.53 - 0.84)**	**0.001**	**0.039**
Postprandial fullness baseline symptom score	6	74.11%	3.267	0.76 (0.56 - 1.02)	0.071	0.554
Early satiation baseline symptom score	8	83.19%%	3.5548	0.68 (0.45 - 1.02)	0.059	0.575
Epigastric pain baseline symptom score	10	86.70%	0.6794	0.87 (0.63 - 1.21)	0.410	0.571
Epigastric burning baseline symptom score	5	88.26%	1.531	0.74 (0.46 - 1.19)	0.216	0.602
Bloating baseline symptom score	8	87.34%	0.0007	1.01 (0.59 - 1.71)	0.979	1.000
Belching baseline symptom score	5	84.85%	1.678	0.62 (0.30 - 1.28)	0.195	0.585
Nausea baseline symptom score	8	82.32%	2.501	0.65 (0.38 - 1.11)	0.114	0.741
